# Inhibition of CDK8 mediator kinase suppresses estrogen dependent transcription and the growth of estrogen receptor positive breast cancer

**DOI:** 10.18632/oncotarget.14894

**Published:** 2017-01-29

**Authors:** Martina S.J. McDermott, Alexander A. Chumanevich, Chang-uk Lim, Jiaxin Liang, Mengqian Chen, Serena Altilia, David Oliver, James M. Rae, Michael Shtutman, Hippokratis Kiaris, Balázs Győrffy, Igor B. Roninson, Eugenia V. Broude

**Affiliations:** ^1^ Department of Drug Discovery and Biomedical Sciences, South Carolina College of Pharmacy, University of South Carolina, Columbia, SC, USA; ^2^ University of Michigan Medical School, Ann Arbor, MI, USA; ^3^ MTA TTK Lendület Cancer Biomarker Research Group, Semmelweis University 2nd Department of Pediatrics, Budapest, Hungary

**Keywords:** CDK8, estrogen receptor, breast cancer, transcription, estrogen independence

## Abstract

Hormone therapy targeting estrogen receptor (ER) is the principal treatment for ER-positive breast cancers. However, many cancers develop resistance to hormone therapy while retaining ER expression. Identifying new druggable mediators of ER function can help to increase the efficacy of ER-targeting drugs. Cyclin-dependent kinase 8 (CDK8) is a Mediator complex-associated transcriptional regulator with oncogenic activities. Expression of CDK8, its paralog CDK19 and their binding partner Cyclin C are negative prognostic markers in breast cancer. Meta-analysis of transcriptome databases revealed an inverse correlation between CDK8 and ERα expression, suggesting that CDK8 could be functionally associated with ER. We have found that CDK8 inhibition by CDK8/19-selective small-molecule kinase inhibitors, by shRNA knockdown or by CRISPR/CAS9 knockout suppresses estrogen-induced transcription in ER-positive breast cancer cells; this effect was exerted downstream of ER. Estrogen addition stimulated the binding of CDK8 to the ER-responsive GREB1 gene promoter and CDK8/19 inhibition reduced estrogen-stimulated association of an elongation-competent phosphorylated form of RNA Polymerase II with GREB1. CDK8/19 inhibitors abrogated the mitogenic effect of estrogen on ER-positive cells and potentiated the growth-inhibitory effects of ER antagonist fulvestrant. Treatment of estrogen-deprived ER-positive breast cancer cells with CDK8/19 inhibitors strongly impeded the development of estrogen independence. *In vivo* treatment with a CDK8/19 inhibitor Senexin B suppressed tumor growth and augmented the effects of fulvestrant in ER-positive breast cancer xenografts. These results identify CDK8 as a novel downstream mediator of ER and suggest the utility of CDK8 inhibitors for ER-positive breast cancer therapy.

## INTRODUCTION

Breast cancer is the most common female cancer and the second leading cause of cancer death in women [1]. Approximately 70% of breast cancers express estrogen receptor alpha (ERα) and are termed ER-positive [2, 3]. ERα, a member of the steroid hormone receptor family, mediates the biological effects of estrogens functioning as a ligand-inducible transcription factor that drives proliferation and survival of ER-positive breast cancer cells [4–6]. Hormone therapies have been developed which specifically inhibit ER signaling including selective estrogen receptor modulators (SERM) such as tamoxifen, selective estrogen receptor down-regulators (SERD) such as fulvestrant, and aromatase inhibitors such as letrozole, which suppress estrogen synthesis [7]. However, many patients with ER-positive cancers do not respond to ER-targeting therapy, or they respond initially but develop progressive disease within 1-2 years due to the development of resistance. Identification of new “druggable” mediators of ER-regulated mitogenic effects could yield critical improvements in the treatment of ER-positive breast cancers [8]. In the present study, we have identified transcription-regulating oncogenic cyclin-dependent kinase CDK8 as a novel druggable mediator of ER signaling.

CDK8 and its closely related paralog CDK19 (80% identity) are transcription-regulating serine/threonine kinases that, unlike better-known members of the CDK family (such as CDK1, CDK2 or CDK4/6), do not mediate cell cycle progression [9]. CDK8 or CDK19, together with their binding partner Cyclin C and MED12 and MED13 proteins, form the CDK module of transcriptional Mediator complex. The CDK module regulates transcription through several different mechanisms, including phosphorylation of the C-terminal domain of RNA Polymerase II in a gene-specific context, which enables the elongation of transcription of newly activated genes [10, 11]. Since CDK8 activity is not generally required for transcription, CDK8 deletion in young adult mouse tissues did not induce any gross or histopathological abnormalities [12] and small-molecule CDK8 kinase inhibitors produced no detectable toxicity at therapeutically active doses [13, 14].

CDK8 activity has been implicated in regulating transcription in several pro-carcinogenic signaling pathways including Wnt/β-catenin [15] and TGFβ/BMP pathways [16]. CDK8 has been identified as an oncogene, which is amplified in many colorectal cancers [15]. CDK8 has also been implicated in melanomagenesis [17], pancreatic cancer [18] and associated with the cancer stem cell phenotype [19]. Our work has identified CDK8/19 as a mediator of damage-induced tumor-promoting paracrine activities of both tumor and normal cells; as a consequence, CDK8/19 inhibitors increase the efficacy of chemotherapy *in vivo* [13].

In the same study, we found that higher expression of CDK8, CDK19 and Cyclin C is associated with shorter relapse-free survival in human breast cancers [13]. More recently, we demonstrated that the same correlations are observed in all principal subtypes of breast cancer and their predictive value is much higher for patients who subsequently underwent systemic adjuvant therapy (either hormonal or chemotherapy), suggesting that CDK8 can impact the failure of systemic treatment in breast cancer. We also found that higher CDK8 protein expression was observed in invasive ductal carcinomas relative to non-malignant mammary tissues [20]. A correlation of CDK8 expression with tumor status, nodal metastasis and stage in breast cancer has also been reported by Xu et al., whose study suggested that CDK8 plays a role in mammary carcinogenesis [21].

We have now discovered that CDK8 acts as a downstream mediator of transcriptional and mitogenic signaling by ER and that inhibition of CDK8 suppresses ER-positive breast cancer cell growth *in vitro* and *in vivo*, potentiates hormone therapy and prevents the emergence of estrogen independence. These findings identify CDK8 as a promising new target to exploit in ER-positive breast cancer treatment.

## RESULTS

### Bioinformatics suggests an association of CDK8 with estrogen receptor signaling

**Figure 1:**
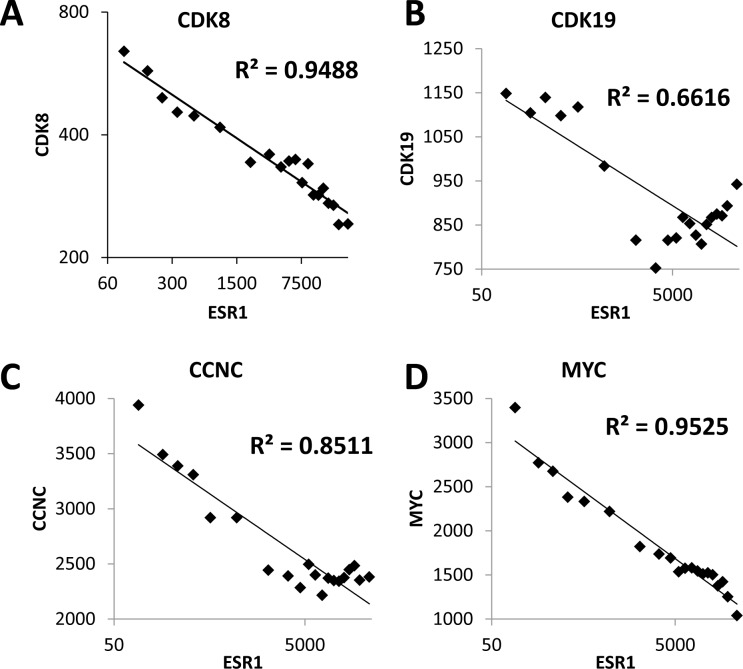
CDK8/19 expression inversely correlates with estrogen receptor expression Correlation between the expression of ESR1 (ERα) and **A**. CDK8, **B**. CDK19, **C**. CCNC and **D**. MYC in 3,491 breast cancers. Each graph shows the average values for 35 bins of 100 samples, arranged by ESR1 expression.

We have extended our previous analysis of CDK8 expression in breast cancer [20] and investigated correlations between CDK8 and ER expression using a microarray database of 3,491 breast cancer patients. Mean CDK8 expression was 1.79-fold higher among 807 ER-negative patients than among 2,036 ER-positive patients (p < 2E-118, t-test). Figure 1A shows a very strong negative correlation between ESR1 (ERα) and CDK8 gene expression among breast cancer samples. Similar inverse correlations with ESR1 were observed for the CDK8 paralog CDK19 and their binding partner Cyclin C (Figure 1B, 1C). These observations, together with a known function of CDK8 as a potentiator of another steroid hormone receptor, the thyroid hormone receptor [22], suggested to us that CDK8/19 could also be acting as a positive effector of ER. If this were the case, an increase in CDK8/19 could enable cells with lower ER levels to utilize estrogen stimulation more efficiently, thus accounting for the inverse correlation. Interestingly, MYC, which has previously been identified as a positive mediator of ER activity [23], also shows an inverse correlation with ESR1 (Figure 1D). Thus, we hypothesized that ER signaling in ER-positive breast cancer could be attenuated through CDK8/19 inhibition.

### Inhibition of CDK8/19 attenuates estrogen-induced transcription

**Figure 2:**
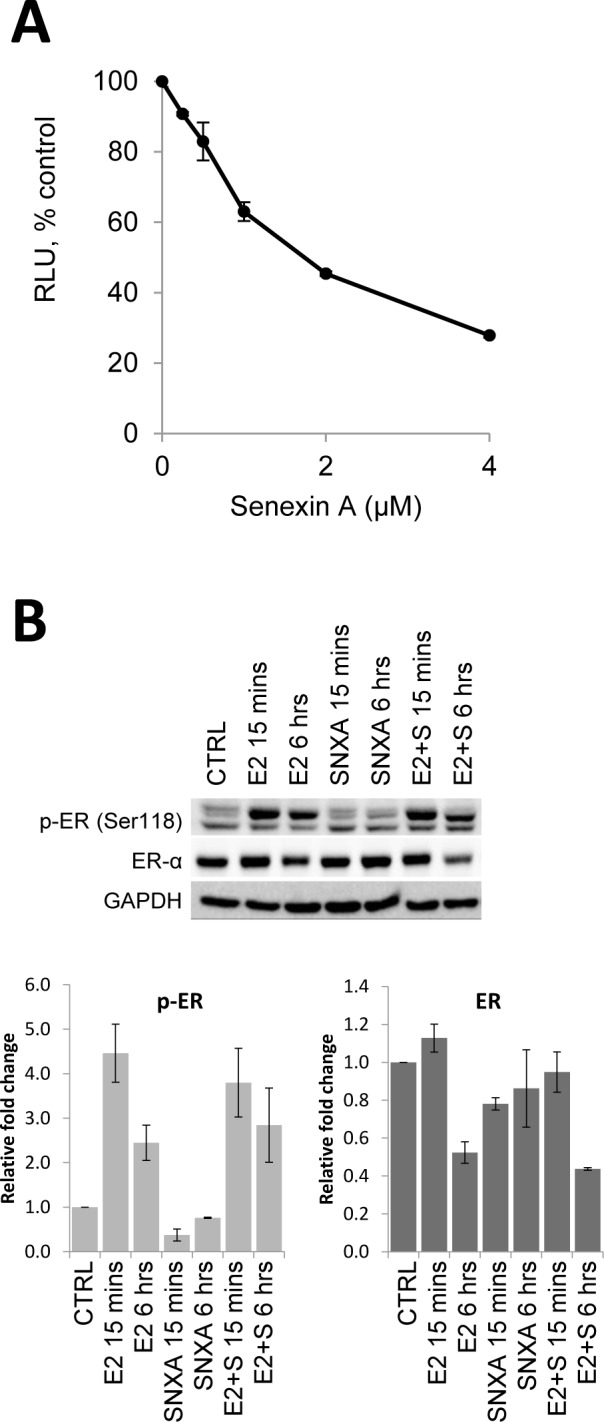
Effects of CDK8/19 inhibition on ER **A**. Luciferase expression from ER-dependent consensus promoter in T47D-ER/Luc reporter cells deprived of estrogen for 72 hours prior to treatment with 10 nM estradiol (E2) and increasing concentrations of Senexin A for 18 hours. RLU refers to relative luciferase units as a percentage of control. **B**. Western blot analysis of phospho-ER (Ser118) and ER expression in T47D-ER/Luc reporter cells deprived of estrogen for 72 hours prior to treatment with 10 nM estradiol (E2) in the presence or absence of 2.5 µM Senexin A for 15 mins and 6 hours. Loading was normalized using GAPDH and densitometry was performed using ImageLab software.

The addition of the selective CDK8/19 inhibitor Senexin A [13] resulted in a dose-dependent decrease in luciferase activity in T47D-ER/Luc cells, expressing firefly luciferase under control of an ER-dependent ERE-containing promoter, suggesting that CDK8/19 potentiates ER-mediated transcription (Figure 2A). Senexin A did not affect ERα protein expression or phosphorylation at S118 (Figure 2B), indicating that CDK8/19 inhibition suppresses ER-mediated transcriptional activity downstream of ER.

**Figure 3:**
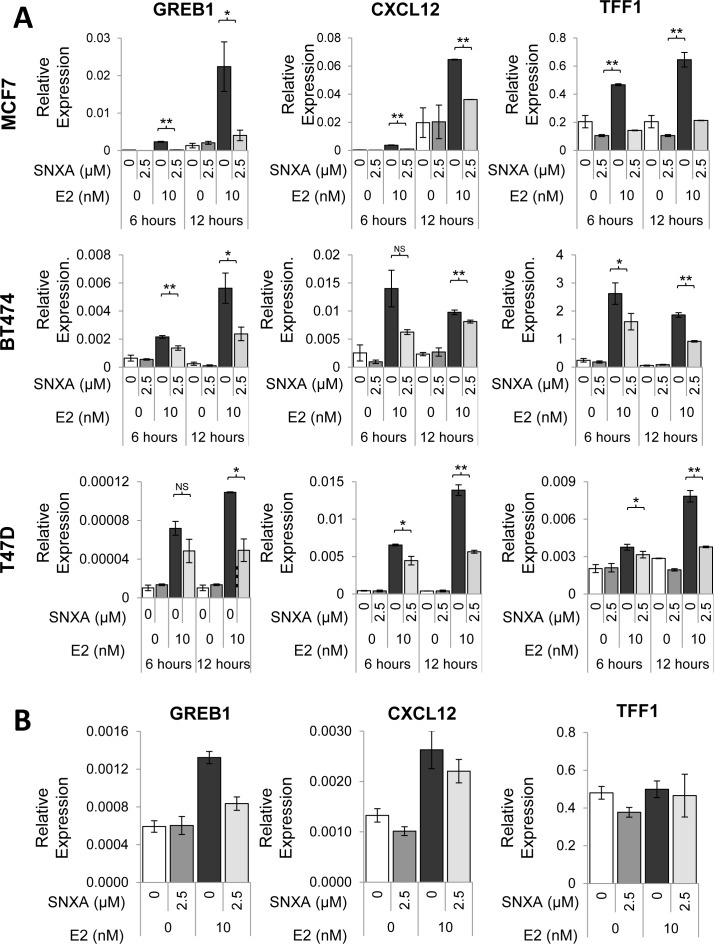
Effects of Senexin A on ER-regulated gene expression **A**. q-PCR analysis of ER-responsive gene expression of GREB1, CXCL12, and TFF1 in estrogen-deprived MCF7, BT474 and T47D-ER/Luc cells treated with E2 (10 nM) and Senexin A (2.5 µM) (SNXA) for 6 and 12 hours. * denotes P < 0.05 and ** denotes P < 0.01. **B**. q-PCR analysis of GREB1, CXCL12, and TFF1 gene expression in MCF7 cells cultured in regular media (without prior estrogen deprivation) following treatment with 10 nM estradiol (E2) and 2.5 µM Senexin A (SNXA) alone and in combination for 12 hr.

Co-administration of Senexin A with estradiol (E2) in ER-positive MCF7 cells prevented estrogen-mediated induction of mRNA of GREB1, CXCL12 and TFF1 (Figure 3A), the three genes that were previously reported to be the most common transcriptional targets of ER in different ER-positive breast cancer cell lines [24]. Similar results were also found in BT474 and T47D-ER/Luc cells (Figure 3A). In contrast to the effect of estrogen addition, Senexin A treatment did not inhibit expression of these three genes in cells growing in estrogen-containing full media, although it decreased the upregulation of GREB1 that was observed upon addition of estradiol to such media (Figure 3B).

### Knockdown or knockout of CDK8 suppresses estrogen-induced gene expression

**Figure 4:**
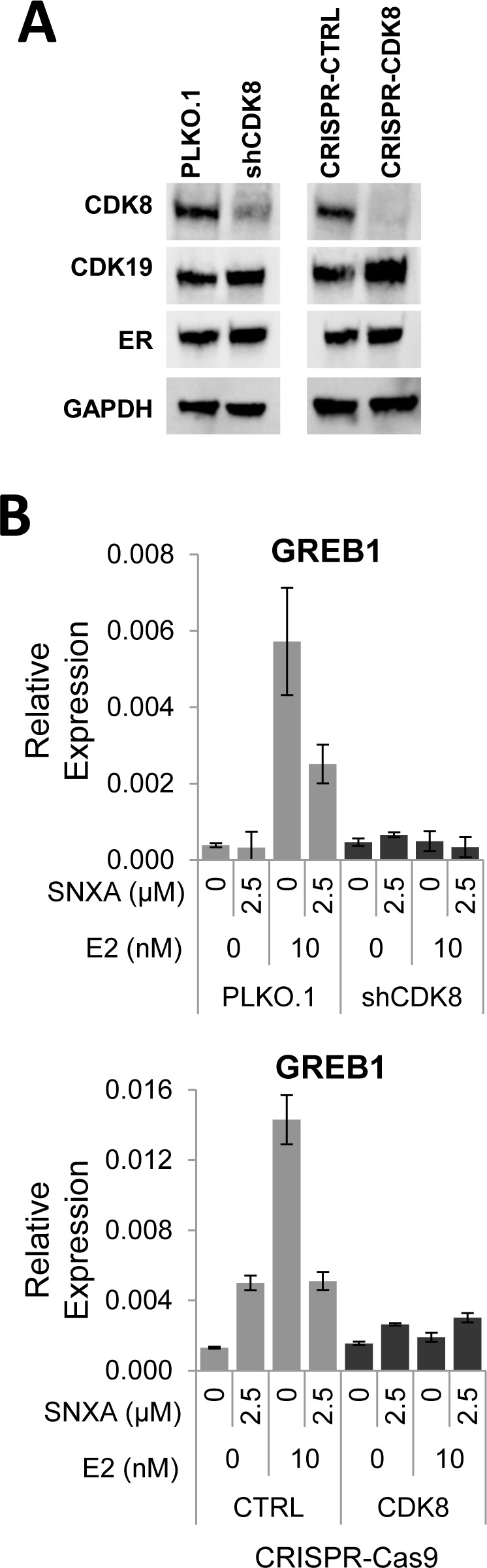
Effects of CDK8 knockdown and knockout on ER-regulated gene expression **A**. shRNA knockdown of CDK8 in BT474 cells (BT474 shCDK8) and CRISPR-Cas9 knockout of CDK8 in BT474 cells (BT474 CRISPR-CDK8) analyzed for protein expression by western blotting. **B**. q-PCR analysis of the effects of E2 on GREB1 gene expression in estrogen-deprived BT474 shCDK8 compared to BT474 PLKO.1 and in BT474 CRISPR-CDK8 compared to BT474 CRISPR-CTRL cells.

Using lentiviral vector-based shRNA against CDK8 in BT474 cells, a stable CDK8 knockdown cell line was established (BT474-shCDK8). Knockdown of CDK8 diminished CDK8 protein expression relative to BT474 PLKO.1 control cells, concomitantly increasing the expression of its paralog CDK19 and without altering ERα expression. Similar effects were also observed in a CRISPR-Cas9 based CDK8 knockout derivative of BT474 cell line (CRISPR-CDK8) (Figure 4A). Gene expression analysis of both BT474-shCDK8 and CRISPR-CDK8 cells revealed that in contrast to vector control-transfected cells, lack of CDK8 renders estradiol incapable of inducing GREB1 and abolishes the effect of Senexin A on GREB1 expression (Figure 4B). These results confirm the data obtained with CDK8/19 kinase inhibitor and suggest that CDK8 alone mediates ER signaling in BT474 cells.

### Inhibition of CDK8/19 has global effects on estrogen-induced transcription

**Figure 5:**
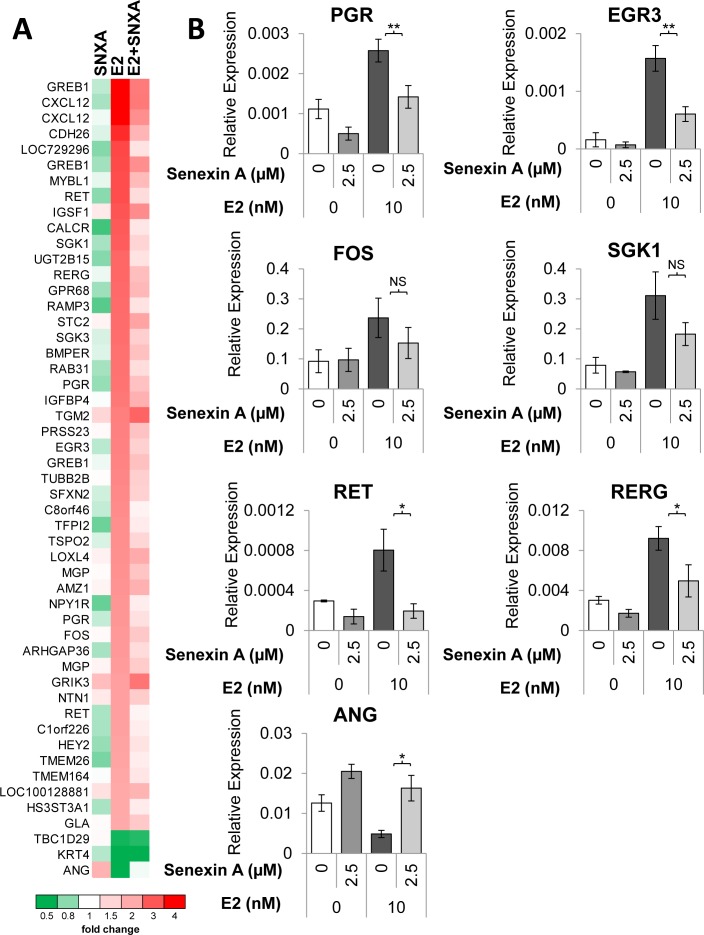
Transcriptomic analysis of the effects of CDK8/19 inhibition on ER signaling **A**. Heatmap of the expression of genes altered > 2-fold (p < 0.05) by estrogen treatment, based on microarray analysis performed on estrogen-deprived MCF7 cells treated with either 10 nM estradiol (E2), 2.5 µM Senexin A (SNXA) or a combination of E2 and Senexin A (E2+SNXA) for 12hr. **B**. q-PCR validation of estrogen regulated genes in MCF7 cells treated with E2 (10 nM) and Senexin A (2.5 µM) for 12 hours. * denotes P < 0.05 and ** denotes P < 0.01.

To determine transcriptomic effects of CDK8/19 inhibition on ER-regulated gene expression we performed microarray analysis of estrogen-deprived MCF7 cells treated with E2 and/or Senexin A. Figure 5A shows a heatmap generated for 45 genes that were altered over 2-fold (p < 0.05) by estrogen treatment; all but three of these genes were induced by estrogen. The effect of estrogen on the majority of those genes (64%; 29/45) was counteracted over 1.5-fold (p < 0.05) by Senexin A addition. Figure 5B shows qRT-PCR validation of the microarray data for PGR, EGR3, FOS, SKG1, RET, and RERG, all of which were induced by estrogen treatment while Senexin A addition significantly decreased this effect (only in the case of PGR, Senexin A inhibited gene expression even without estrogen addition). On the other hand, ANG gene expression was decreased by estrogen treatment and Senexin A counteracted this effect (Figure 5B).

### CDK8 is recruited to GREB1 gene upon estrogen stimulation and mediates the association of elongation-competent phosphorylated form of RNA Polymerase II with GREB1

**Figure 6:**
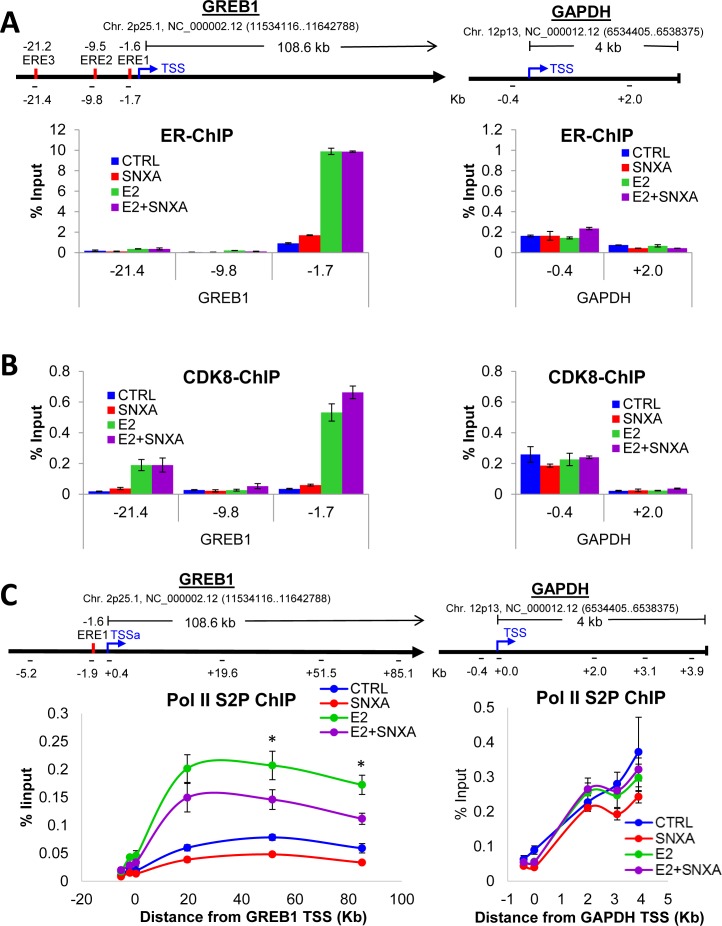
Chromatin immunoprecipitation (ChIP) analysis of ER regulation by CDK8/19 ChIP for ER **A**., CDK8 **B**. and RNA-PolII S2P **C**. was performed on estrogen-deprived MCF7 cells treated with E2 (10 nM) and Senexin A (2.5 µM) for 12 hours. ChIP was followed by q-PCR of different regions of GREB1 and GAPDH. The schematics in **A**. show transcriptional start site (TSS), estrogen receptor binding elements (EREs) and primer binding locations used in **A**. and **B**. (indicated by black bars, with primer positions are listed as the distance from the TSS (Kb)). The schematics in C show the primer binding locations as the distance from the TSS of the respective gene (Kb). * denotes P < 0.05.

Chromatin immunoprecipitation (ChIP) analysis revealed that ERα binding at GREB1 promoter/enhancer regions (close to EREs, as previously reported [25, 26]) is increased following E2 treatment but not significantly affected by CDK8 kinase inhibition (Figure 6A). Remarkably, CDK8 is also strongly recruited to GREB1 promoter/enhancer regions upon the addition of E2; this recruitment is also not significantly affected by Senexin A (Figure 6B; IgG control data are shown in Supplemental Figure 1A). In contrast to its effect on ER-inducible GREB1 gene, E2 had no effect on either ERα or CDK8 binding to a control housekeeping gene, GAPDH (Figure 6A-6B). To test whether decreased E2-dependent gene expression in response to CDK8 inhibition is due to an effect on Pol II CTD phosphorylation at S2 (S2P), which enables the elongation of transcription, we performed ChIP for RNA Pol II S2P on estrogen-deprived MCF7 cells treated with E2 alone or in combination with Senexin A. E2 treatment strongly increased S2P binding along the full length of GREB1, indicating elongation of transcription, while Senexin A significantly decreased the amount of S2P associated with GREB1 in E2-treated cells. In contrast, Senexin A had no significant effect on S2P binding and distribution along the housekeeping GAPDH gene, in agreement with the lack of general CDK8 requirement for transcription (Figure 6C; IgG control data are shown in Supplemental Figure 1B). These results suggest that inhibition of CDK8 kinase activity results in decreased E2-dependent transactivation by inhibiting E2-induced S2 phosphorylation of the Pol II CTD and its associated transcriptional elongation of ER-dependent genes.

### Inhibition of CDK8/19 suppresses the mitogenic effect of estrogen

**Figure 7:**
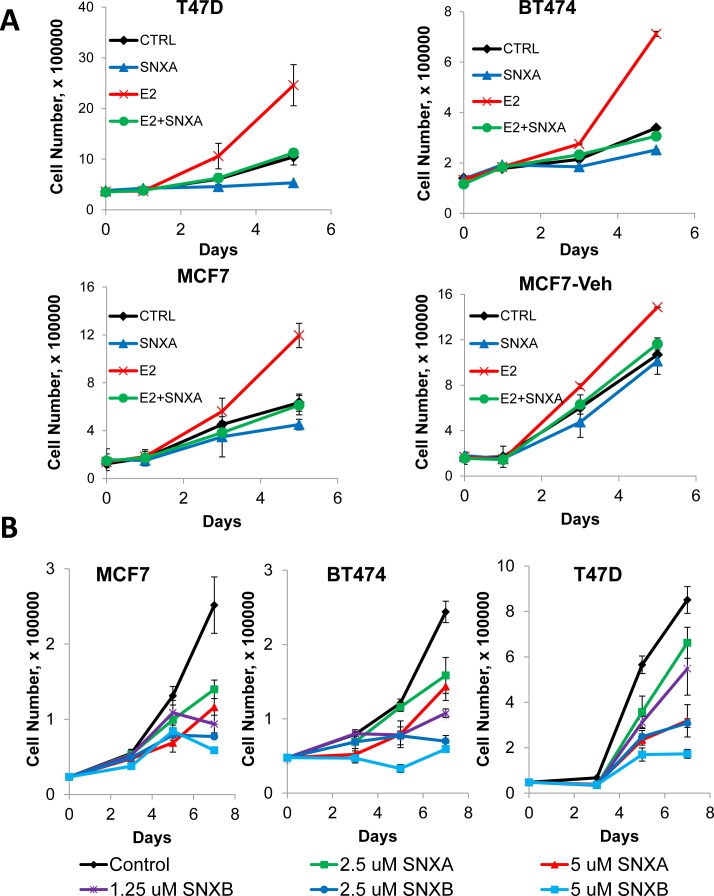
Anti-mitogenic effects of CDK8/19 inhibition in ER-positive breast cancer cells **A**. Effect of 10 nM estradiol (E2) in the presence and absence of 5 µM Senexin A on the growth of estrogen deprived BT474, T47D-ER/Luc, MCF7 and MCF7-Veh cells over 5 days. **B**. Effects of CDK8/19 inhibitors on the growth of MCF7, BT474 and T47D-ER/Luc cells in estrogen-containing media. Cells were treated with a range of Senexin A and Senexin B concentrations and the cell growth was measured by cell counting on days 3, 5 and 7 after treatment.

We asked whether decreased E2-dependent transcription in response to CDK8 inhibition would correspond with a decrease in estrogen-mediated mitogenic signaling. The addition of estradiol to estrogen-depleted MCF7, BT474 and T47D-ER/Luc cells induced robust cell proliferation but this effect of estradiol was abolished by the co-administration of Senexin A (Figure 7A). MCF7-Veh cells are a MCF7 derivative that was adapted to grow in the absence of estrogen and is only weakly stimulated by estrogen [24]. Senexin A had only a weak effect on estrogen-stimulated growth of these cells, reducing their growth to the level of estrogen-untreated cells, in contrast to a much stronger effect on the parental MCF7 cells (Figure 7A), indicating that the anti-mitogenic effects of CDK8/19 inhibition were mediated primarily through ER signaling.

Senexin A also had a dose dependent growth inhibitory effect on ER-positive cells in regular (estrogen-containing) media (Figure 7B). Flow cytometric analysis showed no apparent increase in membrane-permeable (dead) cells after Senexin A treatment (Supplemental Figure 2), indicating that its growth-inhibitory effect was cytostatic rather than cytotoxic. We also evaluated the effects of Senexin B, a newly optimized derivative of Senexin A, which has the same high selectivity for CDK8/19 and is more potent than Senexin A [27]. As expected, Senexin B was more efficient in inhibiting cell growth in the three cell lines compared to Senexin A (Figure 7B).

### CDK8/19 inhibitors prevent the emergence of long-term estrogen independence

**Figure 8:**
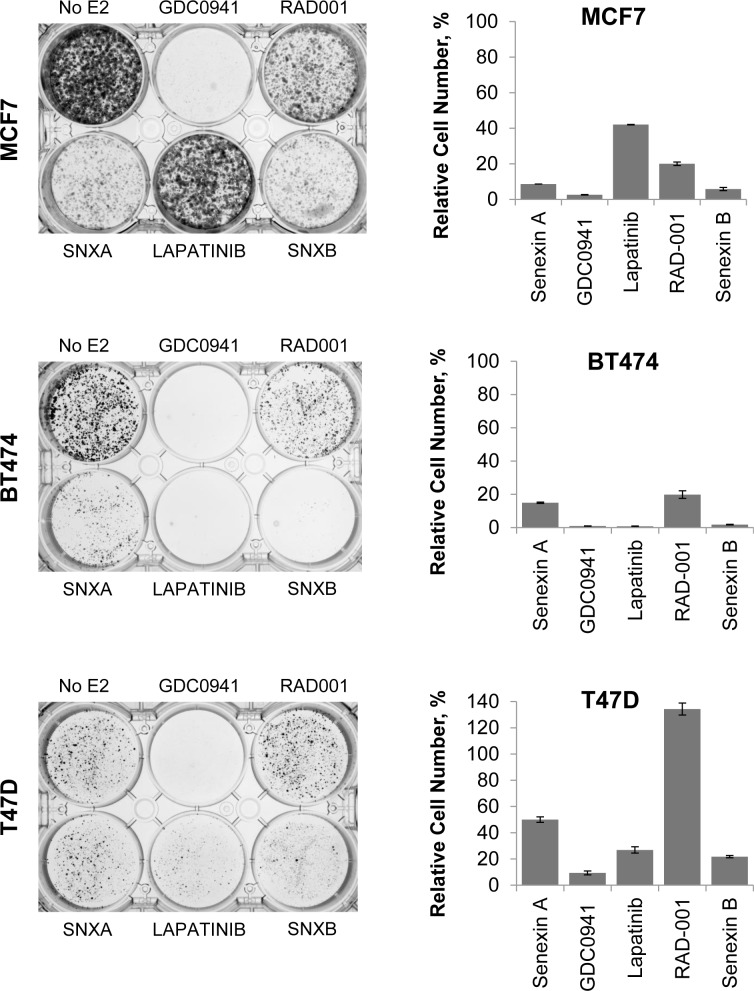
Effects of CDK8/19 inhibition on the emergence of estrogen independent cells Long term growth of MCF7, BT474 and T47D-ER/Luc cells in estrogen deprived media in the presence of Senexin A (5 µM), GDC0941 (1 µM), Lapatinib (1 µM), RAD001 (200 nM) and Senexin B (5 µM). Media and drug were replenished every 3-4 days. Cells were fixed and stained with crystal violet after 15 days for MCF7, 20 days for BT474 and 39 days for T47D-ER/Luc. Additional treated plates of cells were counted and cell counts expressed relative to untreated cells.

Long-term culture of cells in the absence of estrogen mimics the effects of aromatase inhibition in cell culture and exposure of ER-positive cells to long-term estrogen deprivation (LTED) results in the emergence of cells which no longer require estrogen to maintain cell growth [28]. We sought to determine whether combining CDK8/19 inhibition with LTED would slow down or prevent the development of estrogen independence in ER-positive breast cancer cells. To assess this, MCF7, BT474 and T47D-ER/Luc cells were maintained under estrogen depleted conditions and then treated with a range of inhibitors that were previously shown to inhibit the development of estrogen independence [29], including a PI3K inhibitor GDC0941 (pictilisib), a HER2 inhibitor lapatinib and a mTOR inhibitor RAD001 (everolimus), as well as Senexin A and Senexin B. CDK8/19 inhibitors strongly reduced the emergence of estrogen independent cells (Figure 7), with a more potent Senexin B showing a stronger effect than Senexin A. CDK8/19 inhibitors were more effective than mTOR inhibitors or HER2 inhibitors (except for the HER2-positive BT474 cells), and only the PI3K inhibitor, the only cytotoxic agent in this set, was as efficacious as the non-cytotoxic Senexin B (Figure 8). Hence, CDK8/19 inhibition prevents the development of estrogen independence in ER-positive breast cancer cells.

### Inhibition of CDK8/19 suppresses tumor growth and potentiates the effect of fulvestrant *in vitro* and *in vivo*

**Figure 9:**
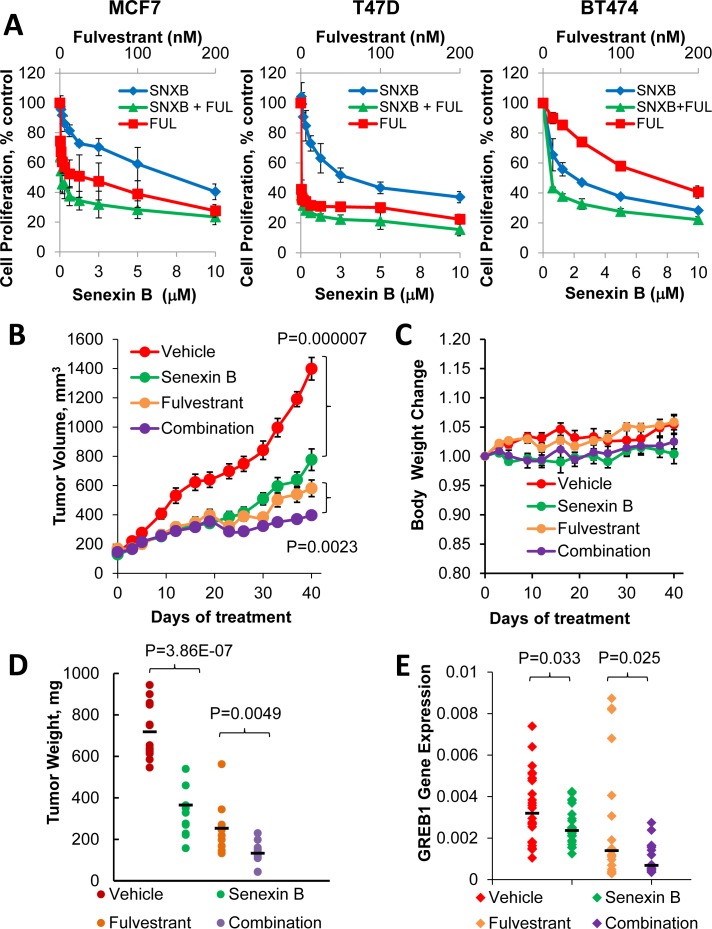
Effects of a CDK8/19 inhibitor and fulvestrant on ER-positive breast cancer cell growth ***in vitro*** and ***in vivo.* A**. Growth inhibitory effects of Senexin B, fulvestrant and a 50:1 mixture of Senexin **B** and fulvestrant in MCF7, BT474 and T47D-ER/Luc. B. Tumor volume changes, **C**. relative mouse body weight changes, and **D**. terminal tumor weights of xenografts generated by subcutaneous injection MCF7 cells in NSG mice (*n* = 11-13 per group), treated with vehicle control, Senexin B (100 mg/kg, twice daily), fulvestrant (5 mg/kg, twice weekly) or a combination of Senexin B and fulvestrant, over 40 days. Data are expressed as Mean ± SEM. **E**. q-PCR analysis of GREB1 gene expression in RNA extracted from MCF7 xenograft tumors.

To examine whether CDK8/19 inhibition may potentiate the effects of ER-inhibiting drugs in estrogen-dependent breast cancer, MCF7, T47D-ER/Luc and BT474 were treated with Senexin A or B, alone or in fixed ratio combinations with a SERD fulvestrant. Senexin A and Senexin B both showed synergy with fulvestrant in MCF7, T47D-ER/Luc and BT474 cells (Figure 9A and Table 1), with the most efficacious effect occurring in BT474 cells when Senexin B was combined with fulvestrant giving a Combination Index (CI) value of 0.16 ± 0.08 (Table 1).

**Table 1:** The effects of fulvestrant and Senexin A or B when combined in a fixed ratio on MCF7, BT474 and T47D-ER/Luc cells measured by MTT assay

Cell Line	Drug 1	Drug 2	drug combination ratio	CI
**MCF7**	Senexin A	Fulvestrant	50 nM : 1 nM	0.39 ± 0.17
	Senexin B	Fulvestrant	50 nM : 1 nM	0.19 ± 0.11
**T47D-ER/Luc**	Senexin A	Fulvestrant	50 nM : 1 nM	0.49 ± 0.36
	Senexin B	Fulvestrant	50 nM : 1 nM	0.20 ± 0.02
**BT474**	Senexin A	Fulvestrant	50 nM : 1 nM	0.44 ± 0.12
	Senexin B	Fulvestrant	50 nM : 1 nM	0.16 ± 0.08

CI values were calculated at 50% effective dose (ED50) using Compusyn software whereby values <1 indicate additive effect, values < 0.7 indicate synergism and values <0.3 indicate strong synergism.

To determine whether the tumor-suppressive effects of CDK8 inhibition and potentiation of fulvestrant activity observed *in vitro* would be recapitulated *in vivo*, we treated mice bearing MCF7 xenograft tumors with a vehicle, Senexin B or fulvestrant alone or with a combination of Senexin B and fulvestrant over a period of 40 days. Both fulvestrant and Senexin B when used alone produced a significant decrease in tumor volume (Figure 9B) without apparent toxicity or significant effects on mouse body weight (Figure 9C). A significant decrease in tumor volume (*p* = 0.0023) (Figure 9B) and terminal tumor weights (*p* = 0.0049) (Figure 9D) between fulvestrant alone and fulvestrant in combination with Senexin B was also observed, indicating that the combination treatment is tolerable and more effective at decreasing tumor growth compared to ER-targeted single agent therapy. Analysis of ER-regulated GREB1 mRNA expression in tumors of different groups indicated that GREB1 expression was significantly suppressed by Senexin B treatment alone (*p* = 0.033). When Senexin B was combined with fulvestrant there was further suppression of GREB1 expression compared to fulvestrant alone (*p* = 0.025) (Figure 9E). These results demonstrate that CDK8/19 inhibition suppresses ER-positive breast cancer growth and potentiates the growth-inhibitory effect of fulvestrant *in vitro* and *in vivo*.

## DISCUSSION

Transcription-regulating kinases CDK7, CDK9 and CDK8/19 have become actively pursued targets in cancer therapy, due in part to their effects on super-enhancers that develop during carcinogenesis and become essential for the survival of tumor cells [14, 30]. A number of groups are developing inhibitors of CDK8/19 Mediator kinase [31], which show numerous effects on oncogenic transcriptional signaling [31, 32] and potentiate the effects of chemotherapeutic drugs by blocking drug-induced transcriptional activation of proteins associated with drug resistance and tumor progression [13]. As single agents, however, CDK8/19 kinase inhibitors showed little or no growth-inhibitory effect in the majority of tested tumor and normal cell types [13]. While the linkage of CDK8 to carcinogenesis was originally discovered in colon cancer [15], CDK8/19 kinase inhibitors did not inhibit the growth of CDK8-overexpressing colon cancer cells [14, 33]. The first evidence for single-agent activity of CDK8/19 inhibitors was reported for a subset of leukemia cell lines, where CDK8/19 inhibition had a strong anti-proliferative effect through hyper-activating super-enhancer-associated genes in such leukemias [14]. We [13, 20] and others [21] have previously shown that CDK8/19 is a negative prognostic marker in breast cancer. In the present study, we have discovered that CDK8 inhibition also inhibits ER signaling and suppresses the growth of ER-positive breast cancer cells, the most common type of breast cancer, *in vitro* and *in vivo*.

Inhibition of ER-mediated transcription by selective small-molecule CDK8/19 inhibitors Senexin A and Senexin B was demonstrated both at the level of the ER-responsive promoter and endogenous gene expression, in three different ER-positive cell lines. Microarray analysis suggested that the effect on ER-regulated genes is global, since the effect of estrogen on most ER-regulated genes was attenuated by CDK8 inhibition. Although Senexin A and Senexin B, like all other reported CDK8 inhibitors, inhibit both CDK8 and CDK19, CDK8 appears to be sufficient for the effect on ER-dependent transcription at least in some ER-positive cell lines, since knockdown or knockout of CDK8 alone was sufficient to suppress both ER-dependent transcription and the effects of CDK8/19 kinase inhibitors on ER-regulated gene expression in BT474 cells. Remarkably, CDK8 inhibition prevented the induction of ER-responsive genes upon estrogen addition to estrogen-depleted cells but did not inhibit such genes in cells maintained in the presence of estrogen. The requirement for CDK8 in de novo gene expression induction but not for sustained expression of genes that are already actively transcribed is in agreement with the previous reports on the effects of CDK8 on other transcription-initiating factors [10, 11].

The effect of CDK8 on transcription appears to be exerted downstream of ER, since CDK8 does not affect the levels of ERα protein, estrogen-induced changes in ERα stability or its phosphorylation at S118, a major but not the sole site of ERα phosphorylation in response to estrogen stimulation [34, 35]. CDK8 inhibition also did not affect the recruitment of ERα to the promoter/enhancer regions of ER-regulated GREB1 gene, indicating that CDK8 exerts its effect downstream of ER. We have found here that CDK8 is recruited, along with ER, to the GREB1 promoter upon estrogen stimulation but this recruitment was also unaffected by CDK8 kinase inhibition. On the other hand, CDK8 inhibition decreased estrogen-stimulated binding of the elongation-competent CTD-phosphorylated S2P form of Pol II, indicating that this phosphorylation provides a mechanism for ER potentiation of CDK8, in agreement with the previously described effects of CDK8 on the transcriptional serum network and HIF1A-induced transcription [10, 11].

Inhibition of ER-mediated transcriptional signaling by CDK8/19 inhibitors was associated with a strong inhibition of the mitogenic effect of estrogen in ER-positive breast cancer cell lines and with cytostatic growth inhibition of ER-positive cells in estrogen-containing media. The anti-mitogenic effect of CDK8/19 inhibitors appears to be mediated primarily or exclusively by ER signaling, since this effect was drastically reduced in a cell line selected for estrogen independence (MCF7-Veh).

Since SERDs act directly on ER and CDK8 acts downstream of it, combining SERDs with CDK8 inhibitors could have a combinatorial or synergistic effect. (In contrast to SERDs, the tumor-suppressive activity of SERMs, such as tamoxifen, involves ER-mediated transcriptional signaling which is modified by this drug [36], and CDK8/19 inhibition could potentially interfere with the effect of SERMs by suppressing ER signaling.) Indeed, a SERD fulvestrant showed a positive interaction with CDK8/19 inhibitors, both *in vitro* and *in vivo*. Interestingly, while the *in vitro* growth-inhibitory effect of fulvestrant alone was much stronger than that of Senexin B alone, the *in vivo* effects of the two compounds were similar, possibly reflecting a role of CDK8/19 in tumor-stromal interactions [13]. Importantly, the combination of Senexin B and fulvestrant showed no apparent toxicity, while producing a stronger tumor-suppressive effect than either drug alone.

We have also found that CDK8/19 inhibitors prevent the development of estrogen independence upon long-term estrogen deprivation (which mimics the effects of aromatase inhibitors) in all three tested ER-positive cell lines. This effect is probably due to the general role of CDK8 in mediating transcriptional reprogramming by enabling the elongation of transcription of newly activated genes [9, 10]. CDK8/19 inhibitors therefore may suppress transcriptional changes associated with the activation of the compensatory signal transduction pathways that complement ER signaling, leading to estrogen independence. The ability to prevent the development of estrogen independence, a major clinical problem in hormone therapy of ER-positive cancers, may offer the greatest therapeutic benefit in the future clinical use of CDK8/19 inhibitors.

## MATERIALS AND METHODS

### Cell culture and reagents

MCF7 and BT474 cells were obtained from ATCC (Manassas, VA, USA); T47D-ER/Luc, which expresses luciferase from an ER-dependent consensus promoter, was obtained from Signosis (Santa Clara, CA, USA); the generation of MCF7-Veh cells was previously described [28]. BT474 cells were maintained in RPMI-1640 (ThermoFisher Scientific, Waltham, MA, USA) with 10% fetal bovine serum (FBS) (Atlanta Biologics, Flowery Branch, GA, USA), 1% penicillin-streptomycin and 2mM L-glutamine (VWR, Radnor, PA, USA). T47D-ER/Luc cells were maintained in the same media as BT474 cells with 75 µg/ml of G418 (Sigma-Aldrich). MCF7 cells were maintained in DMEM-high glucose media (ThermoFisher Scientific) with 10% FBS, 1% penicillin-streptomycin and 2 mM L-glutamine, 1 mM sodium pyruvate (Sigma-Aldrich) and 5 mg insulin (Sigma-Aldrich). For estrogen deprivation (ED), BT474 and T47D-ER/Luc cells were maintained in phenol-red free RPMI-1640 with 10% FBS and MCF7 cells were maintained in phenol-red free DMEM/Hams F12 with 10% FBS (with the same additives as indicated above). After three days, cells were plated in either phenol-red free RPMI-1640 or phenol-red free DMEM/Hams F12 with 10% charcoal-dextran-stripped FBS (ThermoFisher Scientific) (with all additives indicated above) and allowed to grow for 24-72 hours prior to treatment. MCF7-Veh cells were maintained under constant ED conditions. All cell lines were routinely confirmed to be free of Mycoplasma (MycoAlert PLUS mycoplasma detection kit (Lonza, Walkersville, MD, USA) and were authenticated by STR profiling by the University of Arizona Genetic Core (Tucson, AZ, USA) in August 2016. Estradiol (E2) was obtained from Sigma-Aldrich (St Louis, MO, USA), Senexin A and Senexin B dimaleate were from Senex Biotechnology (Columbia, SC, USA), GDC0941 (pictisilib) and RAD001 (everolimus) were from Selleck Chemicals (Houston, TX, USA), Lapatinib was from LC Laboratories (Woburn, MA, USA), Fulvestrant was from ApexBio (Houston, TX, USA).

### Gene expression correlation analysis

Correlation analysis between expression levels of pairs of genes in the Affymetrix microarray dataset of 3,491 breast cancers [37], was carried out using Microsoft Excel. Signal values for 3,491 samples were arranged in ascending order for the first gene and divided into 35 bins of 100 samples (91 samples in the last bin). The median signal values for the first and the second genes were computed for each bin and plotted on log(2) scale. R-squared values for correlation were computed for Power regression.

### Promoter assay

T47D-ER/Luc cells were plated under ED conditions and after 48-72 hours were treated with a range of concentrations of Senexin A, then one hour later with 10 nM E2. Cells were then incubated for a further 18 hours, trypsinized, counted and lysed in lysis buffer (Promega, Madison, WI, USA). Luciferase was measured with Dual-Luciferase E1960 Reporter Assay (Promega) and fluorescence read on FluroStar Optima 960 plate reader using Optima software and normalized by cell number.

### Long term estrogen deprivation assays

MCF7, BT474 and T47D-ER/Luc cells were seeded into duplicate 6-well plates under ED conditions. After 24 hours cells were treated with: Senexin A (5 µM), GDC0941 (pictisilib) (1 µM), Lapatinib (1 µM), RAD001 (everolimus) (200 nM) and Senexin B (5 µM), with DMSO as a vehicle control. Media and drug were replenished every 3-4 days. After 15 days (MCF7), 20 days (BT474) or 39 days (T47D-ER/Luc) one plate was stained with Crystal violet and imaged using a ChemiDoc™ Imaging system. Cells on the remaining plate were trypsinized and counted.

### Mitogenic stimulation assays

Cells were seeded in 6-well plates under ED conditions; after 24 hours an initial cell count was performed (Day 0). Cells were then treated with 10 nM E2 and/or 5 µM Senexin A and cell counts were performed on days 1, 3 and 5 after treatment. Cells were counted in triplicate and cell numbers were plotted relative to control cells.

### Synergy assays

Cells were seeded into 96-well plates and after 24 hours plates were treated with Senexin A or B (0 - 10 µM) and fulvestrant (0 - 200 nM) alone or together in a fixed ratio of 50:1. After 7 days cell proliferation was measured by MTT assay (Sigma-Aldrich).

### Western blotting

Protein (50 µg) was resolved on 8% Express-Plus PAGE gels in Tris-MOPS (SDS) running buffer (GenScript, Piscataway, NJ, USA), transferred to PVDF membranes and incubated at 4°C overnight with primary antibodies: CDK8 (sc-1521, SantaCruz, Santa Cruz, CA, USA), CDK19 (HPA007053, Sigma-Aldrich), ER (sc-543, SantaCruz), phospho-ER Ser118 (sc-101675, SantaCruz) and GAPDH (#5174, Cell Signaling Technology, Danvers, MA, USA) followed by either anti-goat (sc-2020, SantaCruz), anti-rabbit (#31460, ThermoFisher Scientific) or anti-mouse (31430, ThermoFisher Scientific) secondary antibodies. Bands were visualized with Western Lighting Plus ECL detection reagent (Perkin Elmer, Waltham, MA, USA) using ChemiDoc Touch™ (BioRad). Images were analyzed and densitometry performed using ImageLab software (Biorad).

### RNA extraction, reverse transcription, and q-PCR

Cells were seeded in 6-well plates under ED conditions and treated with 10 nM E2 and/or 2.5 µM Senexin A for 6 and 12 hours. Total RNA was extracted using RNAeasy Mini Kit (Qiagen, Hilden, Germany) and 1 µg of total RNA was used to generate cDNA using iScript cDNA synthesis kit (BioRad, Hercules, CA, USA). Gene expression was quantified using iTaq Universal SYBR green super mix on a CFX384 Real time system (BioRad). Primers used for real-time PCR are listed in Supplemental Table 1.

### ChIP assays

Estrogen-deprived MCF7 cells treated with either 10 nM estradiol (E2), 2.5 µM Senexin A or a combination of E2 and Senexin A for 12hr and ChIP assays were performed as previously described [38]. Briefly, cells were fixed with 1% (v/v) formaldehyde, harvested for whole cell lysate preparation and immunoprecipitation was performed with 2 µg of antibodies against CDK8 (sc-1521, SantaCruz), ER-α (sc-543, SantaCruz), RNA-Pol II-S2P (C152000005, Diagenode) and rabbit and goat IgG (sc-2027, sc-2028, Santa Cruz) and enriched DNA was analyzed by q-PCR with ChIP primers (Supplemental Table 2).

### Generation of CDK8 knockout and knockdown cells

BT474 CDK8 CRISPR-Cas9 knockout cells were generated by lentiviral transduction using lentiCRISPR v2 (#52961 Addgene) with the sgRNA targeting human CDK8 (TGCAGCCCTCGTATTCAAACAGG) (designed online using www.crispr.mit.edu and cloned by Viral Vector Core (University of South Carolina School of Medicine)). Parental vector (no-sgRNA) was used a negative control. BT474 CDK8 knockdown cells were generated using pLKO.1 lentiviral virus expressing CDK8 shRNAs (GAACCTGGTATGGGCCATGAG, from Sigma-Aldrich). All virus production was performed in HEK 293FT cells (Invitrogen, Carlsbad, CA, USA). Briefly, at T = 0 cells were transiently transfected with 2 ml of a calcium phosphate precipitation mixture containing pMD2.G (3 μg/ml), psPAX2 (6μg/ml) (Addgene) and 12 μg/ml of pLKO.1 transfer plasmid. At T = 16h, medium was replaced. At T = 24h, 40h and 48h medium was collected, concentrated by overnight centrifugation and re-suspended at a concentration of 1000X in PBS. BT474 cells were infected with MOI = 4 (shCDK8) and MOI = 5 (sgCDK8) followed by 72h puromycin selection (2 µg/ml). Both shRNA knockdown and CRISPR-Cas9 knockout were performed by the Functional Genomics Core (FGC) of the COBRE Center for Targeted Therapeutics, University of South Carolina.

### Microarray analysis

Cells were seeded in biological duplicates under ED conditions, treated with 2.5 µM Senexin A for 24 hours and then treated with 10 nM E2 for 12 hours. Total RNA was extracted as described above and RNA quality was tested using Bioanalyser (Agilent, Santa Clara, CA, USA). Samples were analyzed at the FGC using dual-channel Agilent arrays. Data analysis was performed using limma package in R [39, 40]. Heatmap and unsupervised clustering (using Euclidean distance and Ward clustering) for genes affected at least 2-fold (p < 0.05) by E2 treatment were also performed in R using gplots package [41]. The microarray data in this manuscript is available on the GEO database (GSE93193). A subset of identified genes was validated in biological triplicates by q-PCR.

### *In vivo* xenograft experiments

All animal studies were performed according to guidelines established by the Institutional Animal Care and Use Committee (IACUC) at the University of South Carolina (Columbia, SC). MCF7 cells (1×10^7^ in 50% matrigel) were injected subcutaneously into the flanks of fifty female NSG mice (NOD.Cg-Prkdcscid Il2rgtm1Wjl/SzJ: Stock No: 005557, Jackson Laboratory, Bar Harbor, ME, USA) bearing a 0.36 mg 17β-estradiol pellet (Innovative Research of America, Sarasota, FL, USA). Once tumors reached 100-200 mm^3^ volume, 4 groups of mice (*n* = 11-13) were treated with vehicle, Senexin B dimaleate (100 mg/kg; twice daily, oral gavage in 6.25% 2-Hydroxypropyl-β-cyclodextrin, 1% Dextrose buffer) alone or in combination with fulvestrant (5 mg/mouse; s.c; once/week). Tumor volumes were measured twice weekly with calipers and volumes were calculated using the formula V = W2 x L/2. After 40 days mice were euthanized, tumors were excised and weighed. Tumor fragments measuring 20-30 mg were homogenized in 700 µl of Qiazol reagent and QiaShredder columns, and RNA extracted using chloroform and the RNAeasy Mini Kit (Qiagen), for q-PCR analysis.

### Statistical analysis

All results were presented as mean ± standard deviation of 4-8 parallel assessments. Similar results were obtained from a minimum of two independent experiments. Combination index (CI) values were calculated for MTT combination treatments, using CompuSyn synergy software based on the drug combination principles of Chou-Talalay [42], providing a quantitative definition for additive effect (CI = 1), synergism (CI < 1), and antagonism (CI > 1) in drug combinations. Statistical significance was tested using two-sided Student T-tests and populations were considered significantly different at P < 0.05.

## SUPPLEMENTARY MATERIALS


