# Supplementary material for: Inhibition of CDK8 mediator kinase suppresses estrogen dependent transcription and the growth of estrogen receptor positive breast cancer
Source: Oncotarget. 2017 Jan 29;8(8):12558–75. doi: 10.18632/oncotarget.14894 (PMC5355036; doi:10.18632/oncotarget.14894)
Supplement: Supplementary file 1 [file oncotarget-08-12558-s001.pdf]

Inhibition of CDK8 mediator kinase suppresses estrogen dependent transcription and the growth of estrogen receptor positive breast cancer

Supplementary Material

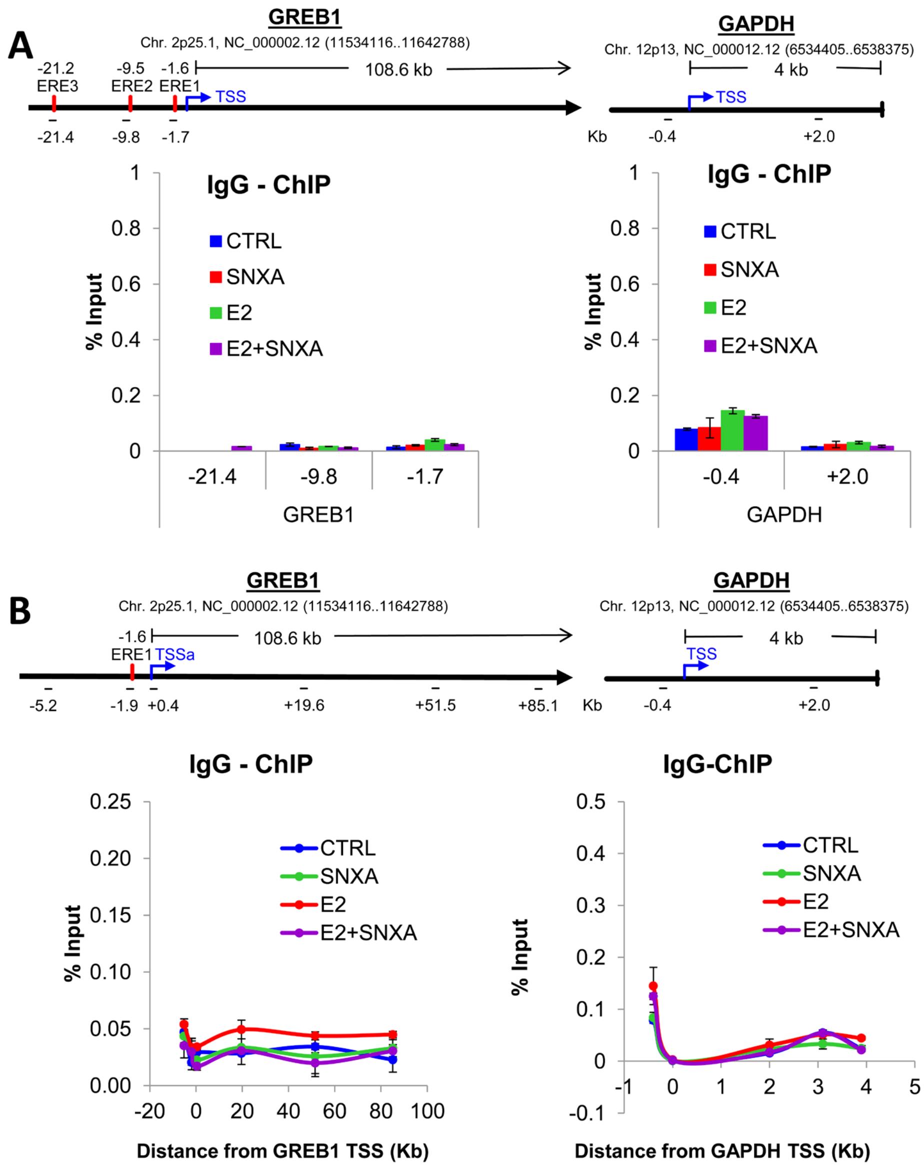

Supplemental Figure 1: A. Chromatin immunoprecipitation (ChIP) for IgG negative control was performed on estrogen-deprived MCF7 cells treated with E2 (10 nM) and Senexin A (2.5 µM) for 12 hours. The transcriptional start site (TSS), estrogen receptor binding elements (EREs) and primer binding locations (indicated by black bars, with primer positions are listed as the distance from the TSS (Kb)) are depicted on a schematic of both human GREB1 and GAPDH genes. B. IgG negative control ChIP was performed on estrogen-deprived MCF7 cells treated with E2 (10 nM) and Senexin A (2.5 µM) for 12 hours. ChIP was followed by q-PCR of multiple regions along GREB1 and GAPDH, schematics showing locations where the primers bind listed as the distance from the TSS of the respective gene (Kb).

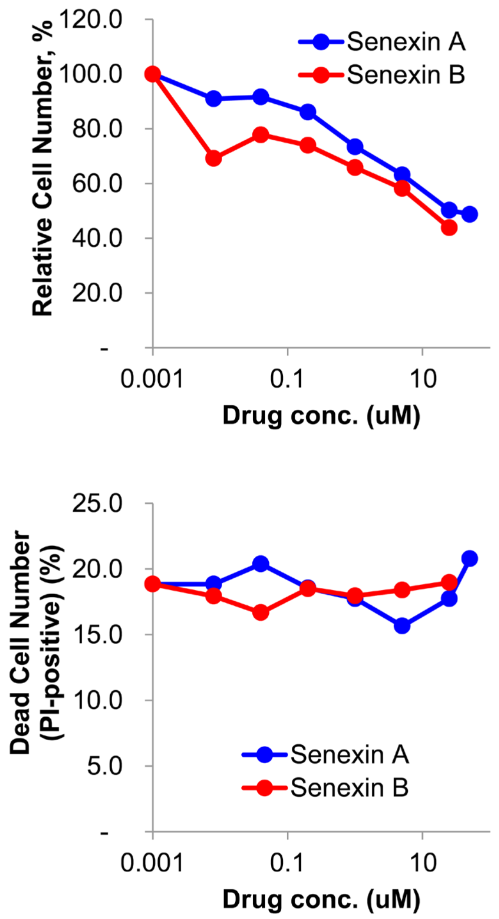

Supplemental Figure 2: Effects of different concentrations of Senexin A and Senexin B over 72 hours in MCF7 cells analyzed by flow cytometry after staining with propidium iodide. Top: cell growth relative to untreated cells, measured by the number of live (PI-negative) cells. Bottom: fraction of dead (PI-positive) cells.

Supplemental Table 1: Primer sequences used for qRT-PCR analysis

Primer		Sequence
GREB1	Forward	AAACAGCTGCAAGGAGGAAG
	Reverse	AACACGTGTGGTGACTGGAG
CXCL12	Forward	CAGAGCTGGGCTCCTACTGT
	Reverse	GCATTGACCCGAAGCTAAAG
TFF1	Forward	AATTCTGTCTTTCACGGGGG
	Reverse	GGAGAACAAGGTGATCTGCG
PGR	Forward	CGATGCAGTCATTTCTTCCA
	Reverse	AATCTGTGGGGATGAAGCAT
EGR3	Forward	TCCATTACAATCAGATGGCTACAGA
	Reverse	TGGTCAGACCGATGTCCATTAC
FOS	Forward	AGGAGGGAGCTGACTGATACACTC
	Reverse	GCAGACTTCTCATCTTCTAGTTGGTCT
SGK1	Forward	GCAGAAGAAGTGTTCTATGCAGT
	Reverse	CCGCTCCGACATAATATGCTT
RET	Forward	AAGCATCCCTCGAGAAGTAGA
	Reverse	CTAGCCGCAGTCCCTCC
RERG	Forward	GTTGCTTGGTGTCGGTAGGT
	Reverse	GGCAATATTTGGGAGAGCAG
ANG	Forward	GCACGAAGACCAACAACAAA
	Reverse	CTTCTTTCCATTGTCCTGCC

Supplemental Table 2: Primer sequences used for CHIP analysis

Distance from TSS		Primer Sequence
GREB1		
-5.2	Forward	TGCAGAGTTGCATTGCAGCAG
	Reverse	CTTTCAAAGTCACTGTGGGCAAA
-1.9	Forward	GGAGCCCTTCATCAGTCAACA
	Reverse	TTCATGAACCTCCCTCGCTC
+0.4	Forward	GCTTTCATACCCCCAGCCTT
	Reverse	CACAAGAGGAGCTCTCACGG
+19.6	Forward	GTGAAGTAGGGAGTGTGCCC
	Reverse	AGCAGCGAAGGGACATGTAG
+51.5	Forward	GGAAGGATGGGCGGAATGAA
	Reverse	CTGCTACCCTCTGACCAACG
+85.1	Forward	CACATCATCTCACTCGGCCA
	Reverse	CTCCGTTCATGGTTCACCGA
-1.7	Forward	GAAGGGCAGAGCTGATAACG
	Reverse	GACCCAGTTGCCACACTTTT
-9.8	Forward	GCCCAGGAGACAGGTTGTAA
	Reverse	TATGACTCTTGGCCCTGTCC
-21.4	Forward	AATCAACCACCAAGCCTCAC
	Reverse	CAGATCCCACAAGGGTCACT
GAPDH		
-0.4	Forward	GCCTTTGCCTGAGCAGTCCG
	Reverse	TCCCCTTTCTTTCTTTCAAAGGCTG
+2.0	Forward	ATGCTGCATTCGCCCTCTTA
	Reverse	GCGCCCAATACGACCAAATC
